# No causal association between insomnia and bladder cancer: a bidirectional two-sample Mendelian randomization study

**DOI:** 10.1186/s40001-024-01920-6

**Published:** 2024-06-08

**Authors:** Lihuan Du, Bohan Wang, Jiaming Wen, Nan Zhang

**Affiliations:** Department of Urology, The Second Affiliated Hospital of Zhejiang University, No. 88 Jiefang Road, Hangzhou, 310009 China

**Keywords:** Insomnia, Bladder cancer, Risk factor, Mendelian randomization, Causal relation

## Abstract

**Background:**

Previous observational studies have indicated a potential link between insomnia and bladder cancer, yet the underlying causal relationship remains uncertain. The current study employed a bidirectional two-sample Mendelian randomization (MR) analysis to investigate this association.

**Methods:**

A two-sample MR analysis was conducted utilizing publicly available summary data from genome-wide association studies (GWAS) on insomnia and bladder cancer. Various regression methods including the inverse variance weighted (IVW), weighted median, MR-Egger, weighted mode, and simple mode methods were employed for the MR analysis. The presence of pleiotropy and heterogeneity in the MR results was also assessed. Furthermore, additional sensitivity tests were performed to mitigate potential biases.

**Results:**

No significant causal relationship was detected between insomnia and bladder cancer using IVW method (OR = 0.761, 95% CI 0.996–1.005; *P* = 0.76). Similarly, the IVW model did not reveal any causal effect of bladder cancer on the risk of insomnia (OR = 1.47, 95% CI 0.772–2.799; *P* = 0.24). Consistent results were obtained from the other four methods employed. There was no evidence of horizontal pleiotropy or heterogeneity in our MR analysis (*P* > 0.05). The sensitivity analyses further supported the reliability of the estimated causal effects.

**Conclusions:**

This study presents no evidence for a causal relationship between insomnia and bladder cancer.

## Introduction

Bladder cancer is a prevalent and potentially fatal malignancy worldwide, with an estimated 17,100 deaths in 2021 in the US [1]. Non-muscle invasive bladder cancer (NMIBC) accounts for ~ 75% of newly diagnosed cases, and a subset of these cases progress to muscle-invasive disease at a rate of 10–15% [2]. Despite notable advancements in therapeutic approaches and tactics in recent years, the survival rate of individuals with muscle-invasive bladder cancer (MIBC) has remained comparatively low [3]. Moreover, NMIBC patients who were unresponsive to Bacillus Calmette–Guérin (BCG) treatment exhibited poorer survival outcomes compared to BCG-responsive patients [4].

The recognized risk factors associated with the onset and progression of bladder cancer include age, tobacco smoking, genetic alterations, dietary patterns, metabolic factors, occupational exposure to certain chemicals such as polycyclic aromatic hydrocarbons, chlorinated hydrocarbons, and diesel exhaust, environmental exposure to substances such as hair dye, and other factors such as cardiovascular disease that remain controversial [5–7].

Insomnia is a prevalent subtype of sleep disorders (SDs) affecting ~ 23–56% of the general population [8]. It has been found to be linked to a diverse range of diseases, including cardiovascular diseases, hypertension, dementia and depression [9–12]. Moreover, insomnia was also identified as a potential risk factor for several types of cancer, including breast, colorectal and gastric cancer [13–15]. Multiple observational studies had also indicated an association between insomnia and the risk of bladder cancer [16, 17]. Another study indicated that insomnia can manifest as a result of bladder cancer treatment, thereby negatively impacting the overall quality of life [18]. However, an alternative observational study demonstrated no significant alterations in health-related quality of life, including insomnia, within the initial 4 years following a diagnosis of NMIBC [19]. In light of the inconsistent findings from these observational studies, further elucidating the relationship between insomnia and bladder cancer is important.

Recently, artificial intelligence, including machine learning, deep learning, and artificial neural networks, has garnered attention for its ability to autonomously learn from extensive datasets and enhance prediction algorithms, particularly in the field of healthcare. This technology shows promise in revolutionizing various aspects of bladder cancer research, such as early detection, precise diagnosis, and personalized treatment strategies [20]. MR, akin to artificial intelligence, is a significant statistical approach employed to establish causal relationships between exposure and outcome by utilizing genetic variants, specifically single-nucleotide polymorphisms (SNPs) [21]. The MR analysis is predicated on three fundamental assumptions: (1) the genetic variant exhibits a robust association with the exposure; (2) the genetic variant does not demonstrate any association with potential confounding factors that may exist between the exposure and the outcome; and (3) the genetic variant solely influences the outcome through the exposure. Therefore, MR analysis, commonly referred to as a "natural randomized controlled trial (RCT)", is less prone to confounding than observational studies and is increasingly employed in the evaluation of causal associations [22].

The objective of this study was to estimate the causal relationship between insomnia and bladder cancer through the implementation of bidirectional two-sample MR analysis.

## Methods

### Summary statistics data for insomnia and bladder *cancer*

The summary statistics for insomnia and bladder cancer were acquired from the IEU Open GWAS project (https://gwas.mrcieu.ac.uk/datasets/). The dataset for bladder cancer (ieu-b-4874) encompasses 1279 cases and 372016 controls of European ancestry, with 9904926 SNPs. The summary statistic for insomnia (ukb-a-13) comprises 336965 individuals of European descent, with 10894596 SNPs. All participants in this study had no sample overlap between the exposure and outcome traits, thereby minimizing the potential bias arising from confounding factors.

### Selection of instrumental variables

The selection of valid instrumental variables (IVs) was guided by three essential assumptions: the relevance assumption, the independence assumption, and the exclusion restriction assumption, as previously described. Specifically, the significance level of IVs for each insomnia trait was established at 5 × 10^–8^, and a total of 30 instrumental SNPs were selected. For bladder cancer, a genome-wide significance level of 5 × 10^–5^ was set, leading to the identification of 95 SNPs as genetic instruments associated with this condition. Furthermore, within the chosen SNPs, a process of linkage disequilibrium (LD) clumping was carried out using a threshold of *r*^2^ < 0.001 and a clumping distance of 10,000 kb. This procedure was used to eliminate any SNPs that were correlated with each other, thus ensuring the independence of the selected IVs. Additionally, SNPs with weak instrumental variable strength (F-statistic greater than 10) were also excluded from consideration.

### Bidirectional Mendelian randomization analysis

Primary two-sample MR analysis was conducted to estimate the ORs and 95% confidence intervals (CIs) utilizing the IVW method, as well as the weighted median, MR-Egger, simple mode, and weighted mode approaches [23]. Initially, each insomnia phenotype was considered as an exposure variable, while the bladder cancer trait served as the outcome variable, aiming to investigate potential causal associations between insomnia and the risk of bladder cancer. Additionally, a reverse MR analysis was performed, wherein each bladder cancer phenotype was considered as an exposure and the insomnia trait was considered an outcome to explore reverse causation between bladder cancer and insomnia risk. Statistical significance was determined at a threshold of *P* < 0.05. Moreover, additional sensitivity analysis was carried out to verify the reliability and stability of the obtained results.

Pleiotropy was evaluated through the utilization of the MR-Egger intercept test, employing Nb Distribution = 1000 [24]. The MR-Egger intercept test investigated the existence of a non-zero intercept to gauge the genetic pleiotropy of IVs, where a significance level of *P* > 0.05 indicated the absence of pleiotropy. Additionally, scatter plots were also generated to examine the potential effect of outliers on the causal correlation.

The assessment of heterogeneity among the SNPs was conducted using Cochrane's *Q* test, with a significance level of *P* > 0.05 suggesting no heterogeneity. Funnel plots were generated to examine the robustness and heterogeneity of the causal correlations. Moreover, the leave-one-out analysis was utilized to evaluate the influence of each SNP on the overall MR estimate and to detect any potential outliers.

All MR analyses were performed using the “TwoSampleMR” package in R4.2.3 (http://www.Rproject.org).

## Results

**Fig. 1:**
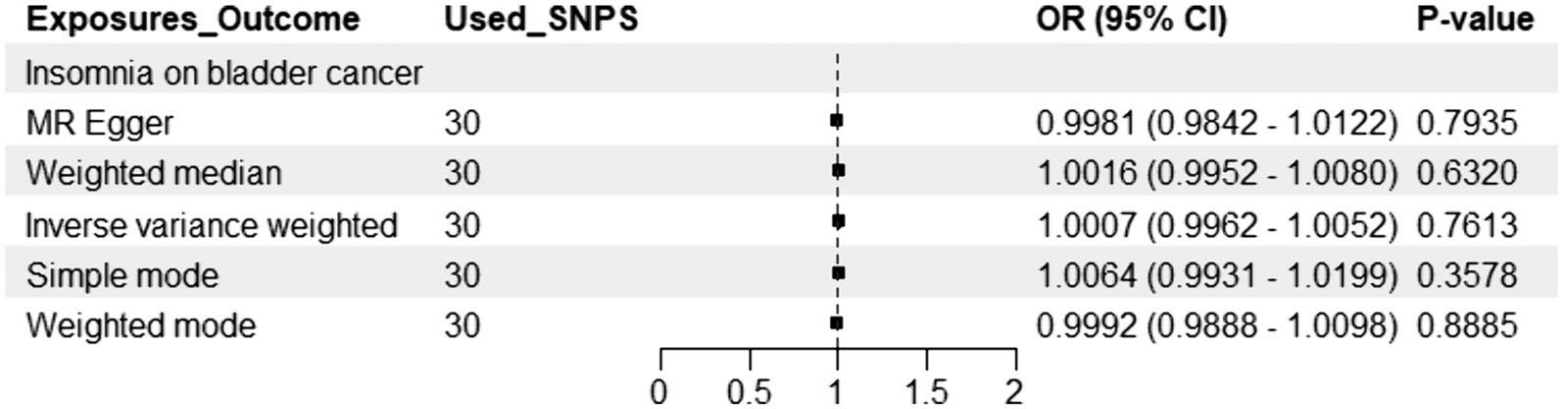
MR estimates of insomnia on the risk of bladder cancer

**Fig. 2:**
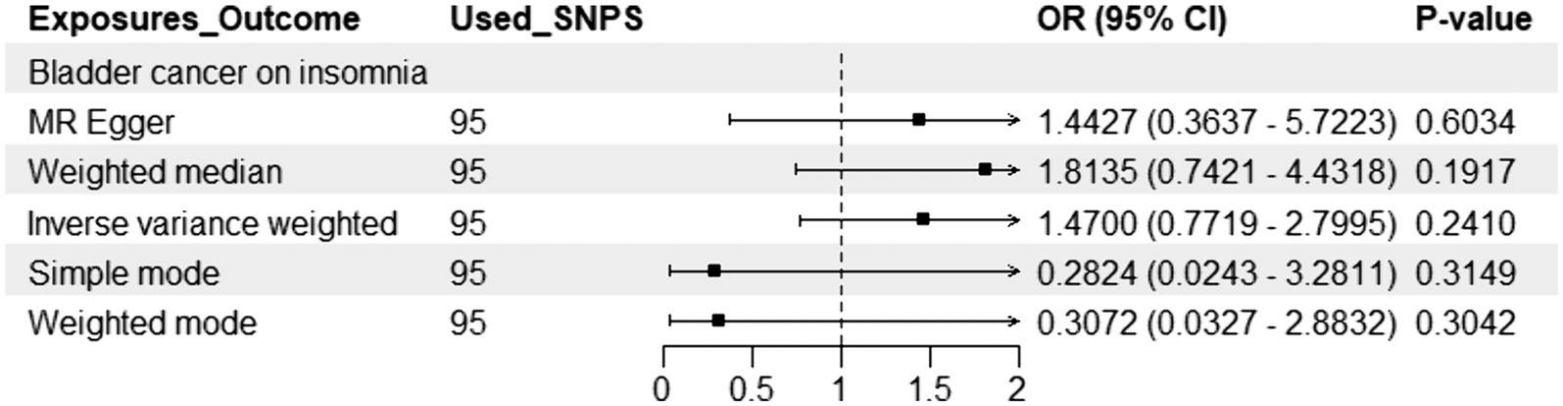
Genetic causal associations between bladder cancer and risk of insomnia

The findings of the present study suggest that there is no causal relationship between insomnia and the risk of bladder cancer. Genetic analysis using the IVW method did not reveal any significant association between insomnia and bladder cancer risk (IVW method: OR = 0.761, 95% CI 0.996–1.005; *P* = 0.76), nor was there any genetic association between bladder cancer and the risk of insomnia (IVW method: OR = 1.47, 95% CI 0.772–2.799; *P* = 0.24). The MR-Egger, weighted median, simple mode and weighted mode methods produced consistent results with IVW analysis (Figs. 1, 2).

**Fig. 3:**
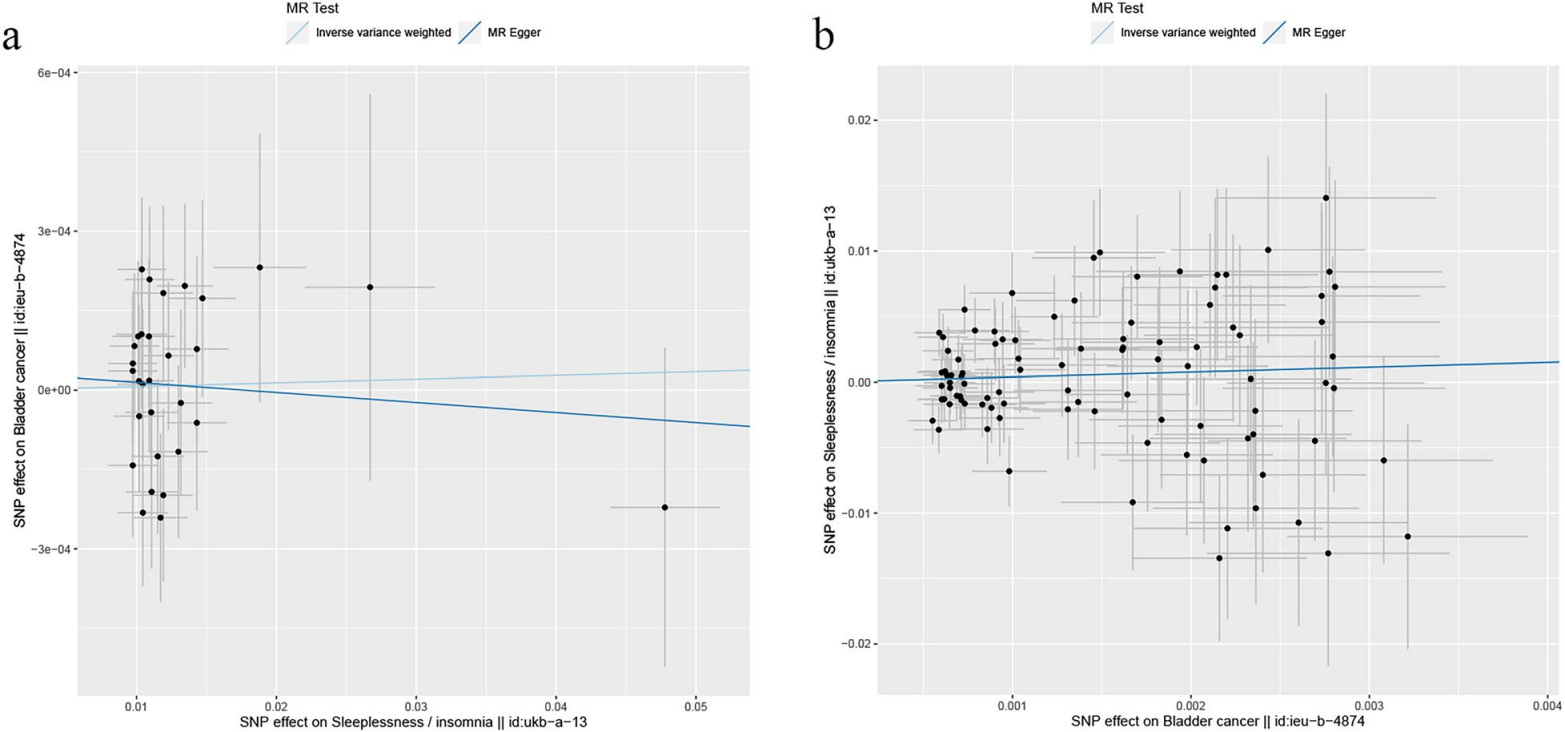
Scatter plot of the MR and reverse MR analysis results between insomnia and bladder cancer

**Fig. 4:**
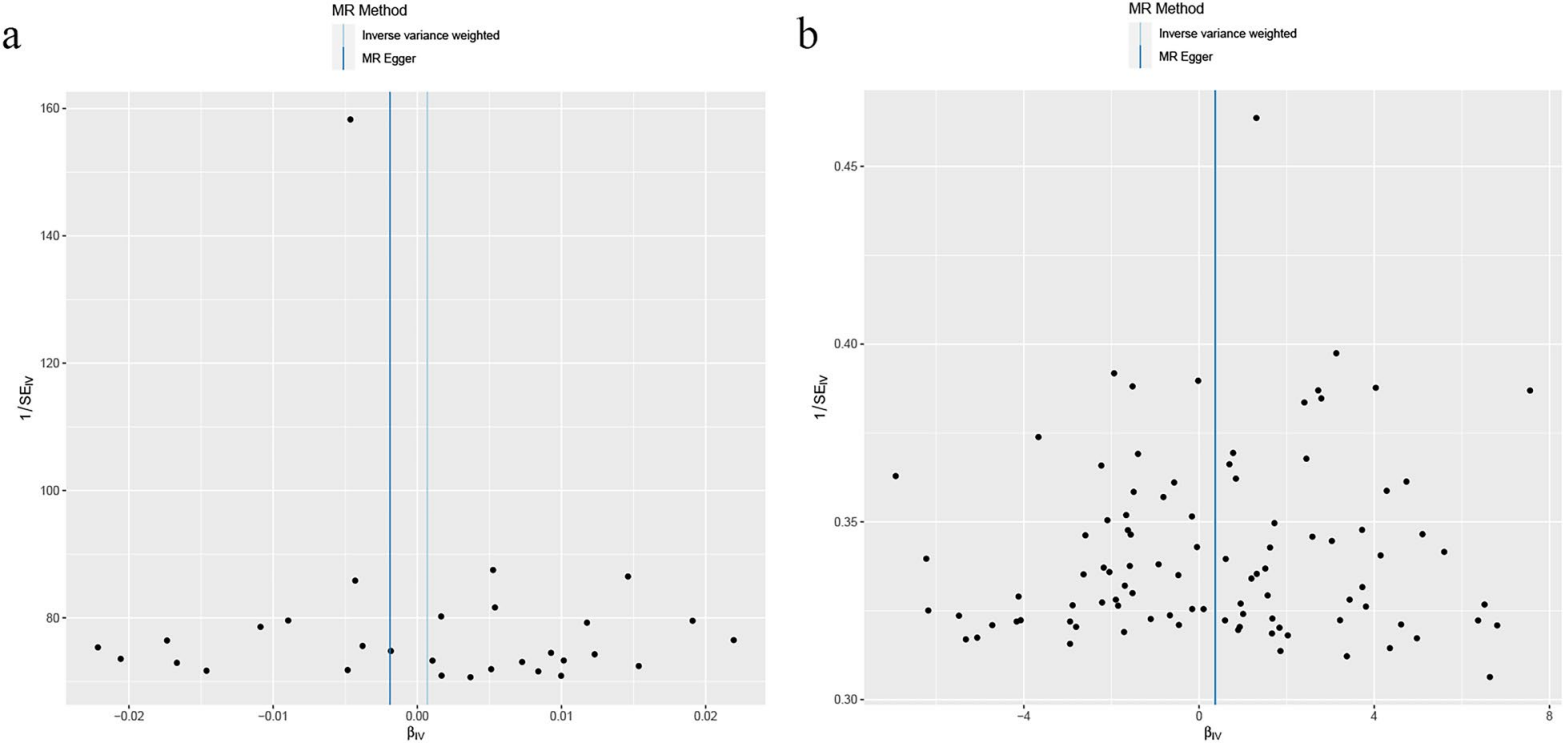
The funnel plot of the causal effect between insomnia and bladder cancer. Individual SNP was delineated in the background

As shown in Table 1, horizontal pleiotropy was not detected in the MR-Egger regression test, and the Cochrane's *Q* statistic (*P* > 0.05) showed no heterogeneity for the selected variants. The scatter plot indicated no potential influence of outliers on the causal correlation (Fig. 3). Furthermore, the funnel plot also showed no evidence for heterogeneity (Fig. 4).

**Table 1:** The heterogeneity and horizontal pleiotropy of MR and reverse MR

Exposure	Outcome	Heterogeneity		Pleiotropy	
		*Q*	*P*	Egger_intercep*t*	*P*
Insomnia	Bladder cancer	23.799	0.692	3.37E−05	0.707
Bladder cancer	Insomnia	112.973	0.078	2.18E−05	0.976

**Fig. 5:**
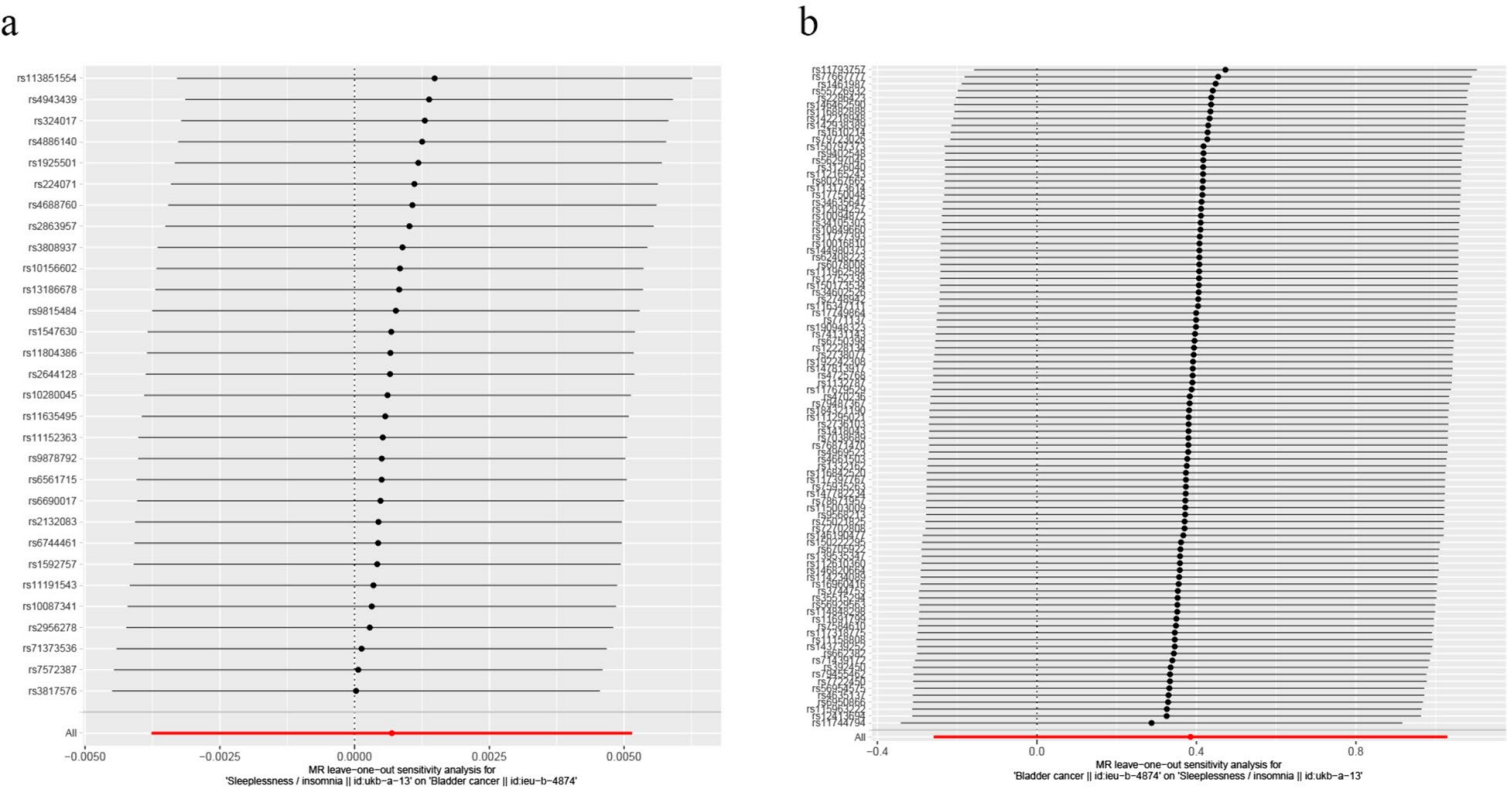
Leave-one-out plots of causal estimates between insomnia and bladder cancer

The sensitivity (leave-one-out) analysis revealed that no potential causality existed after excluding any one SNP (Fig. 5). These results indicated the reliability of the causal effect estimates.

## Discussion

To our knowledge, this is the first MR study to investigate the bidirectional causal association between insomnia and bladder cancer. The findings from our MR study indicate no evidence that insomnia increases the risk of bladder cancer. Furthermore, the reverse MR study failed to demonstrate any substantial evidence supporting a significant association between bladder cancer and the risk of insomnia.

To date, there were two observational studies have provided evidence supporting a positive correlation between insomnia and the risk of bladder cancer. W Li et al. reported that poor overall sleep-quality scores and poor scores for insomnia and snoring status could elevate the risk of several specific-site cancers including breast, uterine or uterine cervical, prostatic, kidney, and bladder cancer in one prospective cohort study that included 78,232 participants [16]. Another retrospective cohort study indicated that HRQoL parameters including pain, fatigue, insomnia, dyspnea and anorexia are significant and independent prognostic factors for outcome (overall survival, time to progressive disease, and time to treatment failure) in patients with locally advanced or metastatic bladder cancer [17].

Recent research has also suggested the possible mechanisms by which sleep disturbances including insomnia might increase the risk of cancer. These mechanisms include the perturbation of circadian rhythms, which play pivotal roles in tumor progression, tumor cell proliferation, and heightened tumor invasion. Additionally, sleep disturbances contribute to a reduction in the secretion of melatonin, a hormone that governs the sleep–wake cycle and plays a fundamental role in safeguarding cells against DNA damage. Furthermore, inflammatory responses triggered by sleep disturbances contribute to uncontrolled cell proliferation [25–28].

Two observational studies have yielded incongruous findings regarding the occurrence of insomnia in patients diagnosed with bladder cancer. Ivy Beeren et al. reported that there were no significant changes in HRQoL including insomnia or social functioning were observed during the first 4 years after NMIBC diagnosis compared with the general population in a multicentre prospective cohort study [19]. In another cross-sectional study, Ahrang Jung et al. demonstrated that 1- to 6-year NMIBC survivors had persistent symptoms, including fatigue, insomnia and financial difficulties [18].

However, it is important to acknowledge that the observational nature of these studies imposes certain limitations, including small sample sizes, selection bias, and susceptibility to confounding factors. Li et al. proposed that the pleiotropy of genetic variants could potentially account for the observed associations [29]. Therefore, the disparities between our study's findings and those of previous cohort studies could potentially be attributed to the aforementioned confounding factors.

However, our MR study also has several limitations. First, the GWASs utilized in our analysis were conducted exclusively on individuals of European descent, thus necessitating further investigation to ascertain the generalizability of our findings to the broader population. Second, despite our efforts to mitigate confounding factors through outlier exclusion and sensitivity analyses, the potential for uncontrolled pleiotropy, heterogeneity, or residual bias cannot be entirely dismissed. This difference may be attributed to the intricate biological mechanisms underlying the functionality of distinct SNPs. Third, larger sample sizes, more sleep disorders such as hypersomnia, snoring and daytime sleepiness, and more advanced approaches are needed to synthesize the results and enhance the statistical power. Finally, the absence of mechanistic studies necessitates further experimental investigations, such as cell function studies and animal studies, to facilitate a comprehensive interpretation of the findings in our study.

## Conclusion

In conclusion, our research indicated the absence of a causal association between insomnia and bladder cancer, which differs from most observational studies. However, larger scale MR studies and more sophisticated methodologies are needed to validate these findings.

## Data Availability

The authors declare that the data supporting the findings of the current study are provided in the article. Any additional inquiries may be directed towards the corresponding author.

## Abbreviations

MR: Mendelian randomization
GWAS: Genome-wide association studies
IVW: Inverse variance weighted
NMIBC: Non-muscle invasive bladder cancer
MIBC: Muscle-invasive bladder cancer
SDs: Sleep disorders
SNPs: Single-nucleotide polymorphisms
RCT: Randomized controlled trial
IVs: Instrumental variables
CIs: Confidence intervals
BCG: Bacillus Calmette–Guérin
